# Folic Acid Is Able to Polarize the Inflammatory Response in LPS Activated Microglia by Regulating Multiple Signaling Pathways

**DOI:** 10.1155/2016/5240127

**Published:** 2016-09-25

**Authors:** Antonia Cianciulli, Rosaria Salvatore, Chiara Porro, Teresa Trotta, Maria Antonietta Panaro

**Affiliations:** ^1^Department of Biosciences, Biotechnologies and Biopharmaceutics, University of Bari, Bari, Italy; ^2^Department of Clinical and Experimental Medicine, University of Foggia, Foggia, Italy

## Abstract

We investigated the ability of folic acid to modulate the inflammatory responses of LPS activated BV-2 microglia cells and the signal transduction pathways involved. To this aim, the BV-2 cell line was exposed to LPS as a proinflammatory response inducer, in presence or absence of various concentrations of folic acid. The production of nitric oxide (NO) was determined by the Griess test. The levels of tumor necrosis factor-alpha (TNF-*α*), interleukin-1 beta (IL-1*β*), and IL-10 were determined by ELISA. Inducible NO synthase (iNOS), nuclear transcription factor-kappa B (NF-*κ*B) p65, MAPKs protein, and suppressors of cytokine signaling (SOCS)1 and SOCS3 were analyzed by western blotting. TNF-*α* and IL-1*β*, as well as iNOS dependent NO production, resulted significantly inhibited by folic acid pretreatment in LPS-activated BV-2 cells. We also observed that folic acid dose-dependently upregulated both SOCS1 and SOCS3 expression in BV-2 cells, leading to an increased expression of the anti-inflammatory cytokine IL-10. Finally, p-I*κ*B*α*, which indirectly reflects NF-*κ*B complex activation, and JNK phosphorylation resulted dose-dependently downregulated by folic acid pretreatment of LPS-activated cells, whereas p38 MAPK phosphorylation resulted significantly upregulated by folic acid treatment. Overall, these results demonstrated that folic acid was able to modulate the inflammatory response in microglia cells, shifting proinflammatory versus anti-inflammatory responses through regulating multiple signaling pathways.

## 1. Introduction

Folic acid belongs to the vitamin B complex. It is vital for red blood cells and for many other cells in the body. Folic acid is also known as folate, folacin, vitamin B9, vitamin M, folvite, acifolic, folcidin, and scientifically as pteroylglutamic acid. The form of folic acid occurring naturally in food is called “folate” and it is a water-soluble vitamin [1].

Previous studies have described anti-inflammatory effects exerted by folic acid, although the mechanisms underlying these actions are still not yet fully clarified [2, 3]. It was reported that combined supplementation of micronutrients, folate, and vitamin B12 has a beneficial effect in reducing inflammation in pregnancy, acting on the levels of inflammatory cytokines [4]. Folate and vitamin B supplementation has been assessed as a potential clinical intervention in vascular disease [5, 6]. In addition, the administration of folic acid to subjects with primary arterial hypertension caused a decreased concentration of inflammation markers (hsCRP, ICAM-1, and VCAM-1) [7]. Kolb and Petrie reported that folate deficiency enhances the proinflammatory cytokine output of RAW264.7 monocytes, suggesting that a folate deficiency may exacerbate cardiovascular disease by augmenting proinflammatory signals in the monocyte-macrophage lineage [8].

Microglia, immune cells resident in the central nervous system (CNS), are responsible for monitoring the CNS environment protecting neurons from invading microorganisms, as well as removing cell degradation products. After recognizing specific pathogen-associated patterns they initiate both innate and adaptive immune responses by producing an array of proinflammatory cytokines, free radicals, and nitric oxide (NO). These microglia responses bring about pathogen clearance and tissue regeneration, but the resulting inflammatory state, if overactivated, may cause neuronal injury. It is now accepted that neuroinflammation is an important contributor to neurodegeneration in various CNS diseases [9].

Suppressors of cytokine signaling (SOCS) proteins are intracellular proteins that inhibit cytokine signaling in a wide variety of cell types [10]. In particular, SOCS proteins have been found to be expressed by immune cells, as well as by cells of the CNS, including microglia [11, 12]. In the context of the inflamed CNS they are involved in preventing excess inflammation, protecting the brain tissue from severe inflammation-induced tissue damage.

The anti-inflammatory effects of folic acid in microglia cells have not yet been well described. Here we reported the effects of folic acid on LPS-induced inflammatory responses of BV-2 microglia cells. The regulatory mechanisms that cause the upregulation of SOCS1 and SOCS3 expression were also investigated.

## 2. Materials and Methods

### 2.1. Cell Cultures and Treatments

The murine-microglia cell line was obtained from the American Type Culture Collection (Manassas, VA, USA). Cells (2 × 10^5^ cells/mL) were plated onto 24-well plates and incubated overnight in Dulbecco's modified Eagle's medium (DMEM; Invitrogen) containing supplementary 10% fetal bovine serum (FBS), 100 units/mL penicillin, 100 *μ*g/mL streptomycin, and 2 mM-glutamine, (Life Technologies-Invitrogen, Milan, Italy) at 37°C in a 5% CO_2_ humidified atmosphere at 37°C. Then, the cells were treated with folic acid (Sigma-Aldrich, Milan, Italy) at final concentrations of 5, 10, 30, 50, 70 *μ*g/mL for 1 hour and then stimulated with LPS (from Escherichia coli 055:B5; Sigma-Aldrich) (1 *μ*g/mL) for the time indicated in the different assays.

### 2.2. Cell Viability Assay

Cell viability was determined by an MTT (Sigma-Aldrich) assay. In brief, BV-2 microglial cells (2 × 10^5^ cells/mL) were plated onto 24-well plates, submitted to folic acid treatment and incubated overnight. Then, culture media were carefully removed and 100 *μ*L of 0.5 mg/mL MTT in cell culture medium was added to each well and incubated for 4 h. Finally, 100 *μ*L of 10% SDS and 0.01 M HCl solution were added to each well to dissolve the formed formazan crystals. The cell viability was calculated according to the following formula: % cell viability = [OD (560 nm) tested compound/OD (560 nm) control cells] × 100. In controls, nontreated cells were cultured in complete medium.

### 2.3. Nitric Oxide Assay

After 24 hours of incubation, supernatants of cultured cells were collected and assayed for NO production using Griess reagent. Briefly, the samples were mixed with an equal volume of Griess reagent (Carlo Erba, Milan, Italy) and then incubated at RT for 10 minutes. The absorbance of supernatants was spectrophotometrically measured at 540 nm and the NO concentration was calculated by extrapolation from a standard curve of sodium nitrite (NaNO_2_). To avoid interference by nitrites possibly present in the medium, a blank of each experiment was performed using the unconditioned medium employed for the microglial culture.

### 2.4. Reverse Transcriptase-Polymerase Chain Reaction (RT-PCR) and Quantitative Real-Time PCR Analyses

Total RNA was isolated from cells using Trizol reagent according to the manufacturer's protocol. The mRNA levels of various genes were quantified using the SYBR Green QuantiTect RTPCR Kit (Roche, South San Francisco, CA, USA). GAPDH was used as endogenous reference. Data were analyzed using the relative standard curve method according to the manufacturer's protocol. The mean value of each gene after GAPDH normalization at the time point showing the highest expression was used as calibrator to determine the relative levels of TNF-*α*, IL-1*β*, iNOS, IL-10, Arginase (ARG)-1, and CD206 at different time points. The primer sequences for the tested genes are reported in Table 1.

### 2.5. Electrophoresis and Immunoblotting

After treatment as previously described, cells were washed twice in PBS, detached with ice-cold PBS, collected, and centrifuged at 600 g for 10 min. The supernatants were then removed and the pellet was harvested and lysed by ice-cold lysis buffer [1% (v/v) Triton X-100, 20 mM Tris-HCl, 137 mM NaCl, 10% (v/v) glycerol, 2 mM EDTA, 1 mM phenylmethylsulfonyl fluoride (PMSF), 20 *μ*M leupeptin hemisulfate salt, and 0.2 U/mL aprotinin (all from Sigma Aldrich)] for 30 min on an ice-bath. The protein concentration in the supernatant was spectrophotometrically determined by Bradford's protein assay and the lysate was subjected to SDS-PAGE (NuPage Electrophoresis System, Invitrogen). After electrophoresis, the resolved proteins were transferred from the gel to nitrocellulose membranes using iBlot Dry Blotting System A (Life-Technologies). Antibodies directed against *β*-actin, iNOS, ERK 1/2, p-Erk 1/2, p-I*κ*B*α*, I*κ*B*α*, p38 MAPK, p-p38 MAPK, JNK, p-JNK, SOCS1, and SOCS3 were all obtained from Santa Cruz Biotechnology, Inc. (Santa Cruz, Heidelberg, Germany) (diluted 1 : 1000 for p-I*κ*B*α*, ERK 1/2, p-Erk 1/2, p-p38 MAPK, p38 MAPK, JNK, p-JNK; 1 : 100 for iNOS; 1 : 500 for SOCS1 and SOCS3, and 1 : 10,000 for *β*-actin). Membranes were incubated with the secondary antibody horseradish peroxidase (HRP) conjugate (Santa Cruz Biotechnology) diluted 1 : 2000, for 60 min at room temperature in the dark on a shaker. Finally, bands were visualized by the chemiluminescent method (BioRad Laboratories, Hercules, CA, USA). The *β*-actin protein level was used as protein loading control in western blotting. The bands obtained after immunoblotting were submitted to densitometric analysis using ID Image Analysis Software (Kodak Digital Science), expressing results expressed as arbitrary units.

### 2.6. ELISA Assay

For ELISA assay, the supernatant of cultured cells stored at −70°C was used to measure the cytokines release. The concentrations of IL-1*β*, IL-10, and TNF-*α* were determined by ELISA according to the manufacturer's protocols (R&D System Inc., Minneapolis, MN).

### 2.7. Statistical Analysis

Student's *t*-test and analysis of variance (one-way ANOVA) were performed on the results of at least five independent biological replicates. Values of *p* < 0.05 were considered statistically significant.

## 3. Results

### 3.1. Effect of Folate Treatment on Cell Viability

None of the 5–50 *μ*g/mL range concentrations of folic acid tested affected the viability of BV-2 microglial cells, although folate 70 *μ*g/mL resulted to be cytotoxic (Table 2). Thus, for the study of microglial cells functional responses we used concentrations ranging from 5–50 *μ*g/mL, as reported in literature [13].

### 3.2. Effect of Folate Treatment on NO Production and iNOS Expression

**Figure 1:**
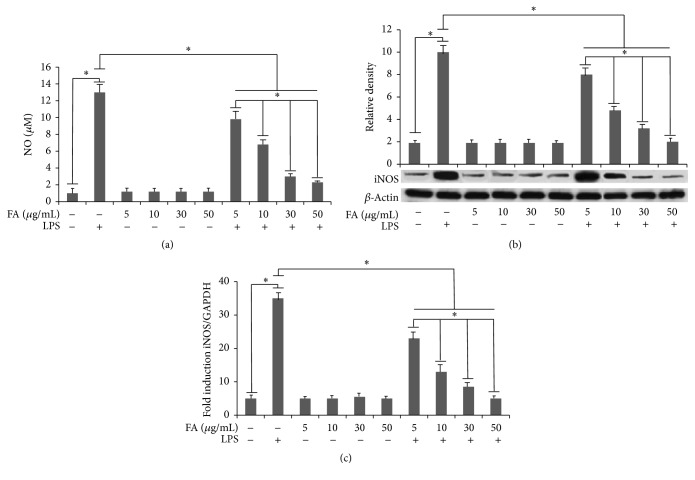
(a) Effects of folic acid on LPS-induced NO production in BV-2 cells. Cells were preincubated in medium containing various concentrations of folic acid (FA) (5, 10, 30, and 50 *μ*g/mL) for 1 h and then treated with LPS (1 *μ*g/mL) for 24 h. The amount of nitrite in the medium was measured by Griess reaction. (b) iNOS expression in BV-2 cells. Total cellular protein samples were collected and examined by western blotting. Protein levels were determined by measuring immunoblot band intensities by scanning densitometry. (c) iNOS mRNA expression determined by real-time RT-PCR. BV-2 microglial cells were incubated for 6 h with LPS alone, or after 1 h of pretreatment with different amounts of folates. Experimental treatments were analyzed in triplicate and data are expressed as the mean ± SD of five independent experiments. ^*∗*^ *p* < 0.05.

Folate treatment significantly lowered the LPS-induced NO release by microglia cells (Figure 1(a)). We also found that folate significantly and dose-dependently attenuated NO production after 24 h LPS treatment, reaching a maximal inhibition at 50 *μ*g/mL. Interestingly, iNOS expression was significantly and dose-dependently downregulated in the presence of folate (Figure 1(b)). The qPCR analysis confirmed these results also for the mRNA levels of this inflammatory mediator (Figure 1(c)). Taken together, these results indicate that folate treatment suppresses the release of NO in LPS-stimulated BV-2 microglial cells by modulating iNOS expression also at transcriptional levels.

### 3.3. Effect of Folic Acid on LPS-Induced Cytokines Expression and Microglia Activation

**Figure 2:**
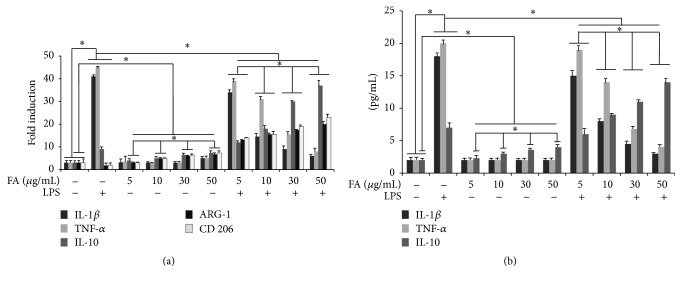
Effect of folic acid on cytokines production and microglia activation in LPS-stimulated BV-2 cells. (a) Proinflammatory cytokine and microglia activation markers mRNA expression determined by real-time RT-PCR. BV-2 microglial cells were incubated for 6 h with LPS alone or after 1 h of pretreatment with different amounts of folates. (b) ELISA assay. Cells were treated with the indicated doses of folic acid for 1 h before LPS (1 *μ*g/mL) treatment. Supernatants were collected at 24 h after LPS treatment and the amounts of IL1-*β*, IL-10, and TNF-*α* were measured. This assay was performed in triplicate and data are expressed as the mean ± SD of five independent experiments. ^*∗*^ *p* < 0.05.

RT-PCR was performed to determine whether folic acid regulates proinflammatory cytokine and microglia activation markers expression at transcriptional level. As shown in Figure 2(a) we demonstrated a marked increase in TNF-*α* and IL-1*β* mRNA in BV-2 microglial cells after 6 h of LPS treatment. By contrast a significant reduction of ARG-1 and CD206 microglia activation markers was evidenced in LPS treated BV-2 microglial cells. No effect of treatment with folic acid alone on proinflammatory gene expression was observed in cells, proinflammatory cytokines mRNA levels being similar in all tested concentrations (5–50 *μ*g/mL) and comparable to the levels detected in control cells. Interestingly, we observed that mRNA expression of proinflammatory cytokines was significantly decreased in LPS-treated cells after pretreatment with folic acid in comparison to microglial cells stimulated with LPS alone, suggesting that, in LPS-activated cells, folic acid was able to downregulate proinflammatory cytokine mRNA expression, in a dose-dependent manner, reaching a maximal reduction at 50 *μ*g/mL (Figure 2(a)).

ELISA was performed in order to investigate whether folate regulates proinflammatory cytokine expression. Folic acid treatment alone had no effect on the production of IL-1*β* and TNF-*α* in BV-2 microglia. The increased levels of IL-1*β* and TNF-*α* levels in supernatants obtained from 24 h LPS-stimulated BV-2 microglia resulted significantly reduced after pretreatment with folic acid in a dose-dependent manner, as shown in Figure 2(b).

Concerning the effect of folate on the regulation of the anti-inflammatory cytokine, IL-10, in LPS treated cells we observed a significant increase of IL-10, while folate pretreatment was able to upregulate this expression. Interestingly, IL-10 levels resulted significantly increased in folate pretreated cells in terms of both transcript and protein and this regulation was dose-dependent, as reported in Figures 2(a) and 2(b). Same results were revealed for the ARG-1 and CD206 mRNA of LPS treated BV-2 microglial cells pre-treated with folic acid (Figure 2(a)).

### 3.4. Effect of Folate on the Signalling Pathways Evoked by LPS-Activated BV-2 Cells

**Figure 3:**
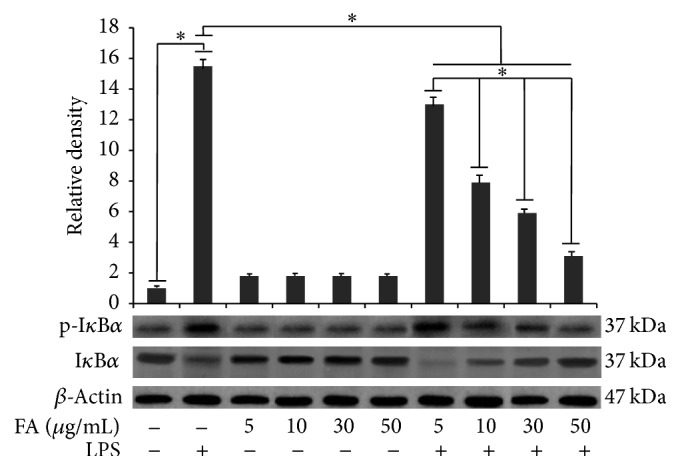
Effects of folic acid on the LPS-induced phosphorylation of I*κ*B*α*. Cells were treated with the indicated doses of folic acid (5, 10, 30, and 50 *μ*g/mL) for 1 h before LPS (1 *μ*g/mL) treatment for 12 h. Total cell lysates were prepared for western blot analysis for the content of p-I*κ*B*α* protein in BV-2 cells. Protein levels were determined by measuring immunoblot band intensities by scanning densitometry. Data represent the means ± S.D. of five separate experiments. ^*∗*^ *p* < 0.05.

**Figure 4:**
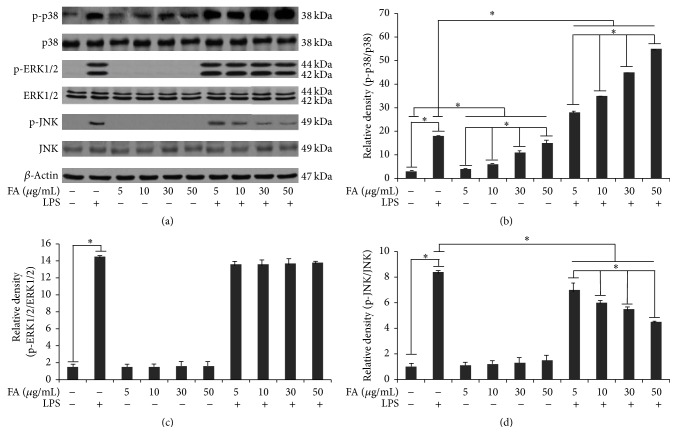
Signaling pathways evoked by LPS-activated BV-2 cells in folic acid-treated cells. Cells were treated with the indicated doses of folic acid (5, 10, 30, and 50 *μ*g/mL) for 1 h before 12 h LPS (1 *μ*g/mL) treatment. (a) Total cell lysates were prepared for a western blot analysis of the content of p-38 protein, p-ERK 1/2, and p-JNK in BV-2 cells. Protein levels were determined by measuring immunoblot band intensities by scanning densitometry: (b) p38 and p-p38; (c) ERK 1/2 p-ERK 1/2; (d) JNK and p-JNK. Data represent the means ± S.D. of five separate experiments. ^*∗*^ *p* < 0.05.

The role played by folic acid in cell signalling induced by 12 h LPS stimulation was also investigated. For this purpose, we firstly investigated NF-*κ*B activation. Since the degradation of I*κ*B-*α* is essential for the nuclear translocation of NF-*κ*B p65, we determined the effect of folic acid on LPS-induced phosphorylation of the I*κ*B-*α* protein, by western blotting. Cells stimulated with LPS exhibited a significantly increased p-I*κ*B*α* expression in comparison to controls (Figure 3). Densitometric analysis revealed a faint phosphorylation of I*κ*B*α* in unstimulated cells (Figure 3). Pretreatment with folic acid significantly dose-dependently reduced p-I*κ*B-*α* in LPS-activated cells, as reported in Figure 3. In addition to the NF-kB pathway, the effect of folic acid on the activation of the ERK 1/2, JNK, and p38 pathways was examined in LPS-activated microglia cells using western blotting analysis. As shown in Figures 4(a) and 4(b), folic acid significantly increased LPS-induced phosphorylation of p38 kinase in BV-2 cells in a concentration-dependent manner, whereas JNK phosphorylation was dose-dependently reduced by folic acid (Figures 4(a) and 4(d)). Conversely, ERK 1/2 kinases phosphorylation was not affected by folic acid treatment (Figures 4(a) and 4(c)). Finally, the amounts of total ERK 1/2, JNK, and p38 were unaffected by LPS in combination with folic acid treatment.

These results suggest that folic acid could modulate LPS-induced inflammatory mediatory production through regulating multiple signalling pathways.

### 3.5. SB203580 Treatment Downregulates IL-10 Upregulation by Folic Acid

**Figure 5:**
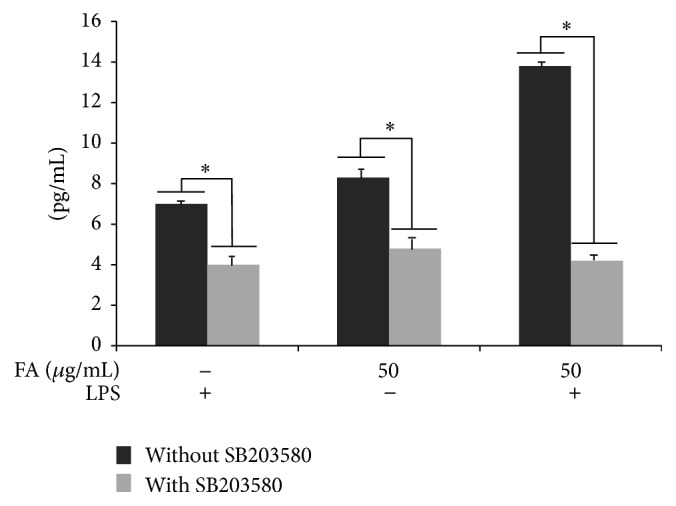
IL-10 production induced by folic acid (50 *μ*g/mL) activity is downregulated by p38 inhibition. BV-2 cells were incubated with 10 *μ*M of p38 pharmacological inhibitor, SB203580, 1 hour before LPS treatment in the presence or absence of folic acid. The supernatants were collected after 24 h LPS treatment and assayed for IL-10 release. This assay was performed in triplicate and data are expressed as the mean ± SD of five independent experiments. ^*∗*^ *p* < 0.05.

To confirm the role of p38 on IL-10 overproduction by 24 h LPS activated cells, pretreated with folic acid (50 *μ*g/mL), the cytokine expression levels were analyzed by ELISA. As shown in Figure 5, treatment with the p38 pharmacological inhibitor, SB203580 (10 *μ*M), induced a significant decrease of IL-10 levels with respect to both LPS-stimulated cells in presence of folate and cells treated with folic acid alone, highlighting the important role of p38 kinase in the upregulation of IL-10 production by folic acid treatment.

### 3.6. Modulation of SOCS1 and SOCS3 in BV-2 Treated with Folic Acid

**Figure 6:**
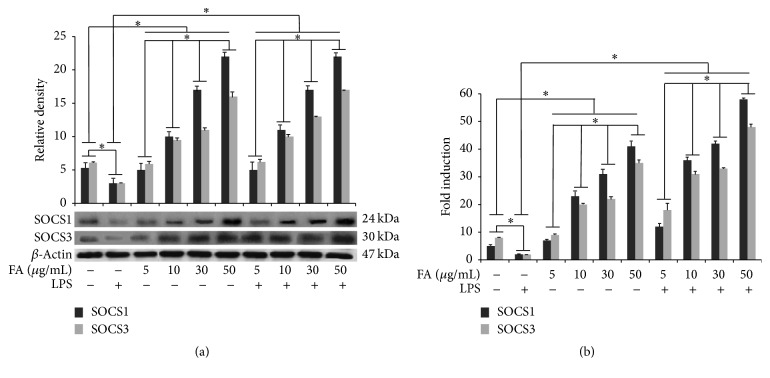
Modulation of SOCS proteins in BV-2 treated with folic acid. (a) Cells were treated with the indicated doses of folic acid (5, 10, 30, and 50 *μ*g/mL) for 1 h before LPS treatment. Total cell lysates were prepared for western blot analysis for the content of SOCS1 and SOCS3 in BV-2 cells. Protein levels were determined by measuring immunoblot band intensities by scanning densitometry. (b) mRNA expression determined by real-time RT-PCR. BV-2 microglial cells were incubated for 6 h with LPS alone or after 1 h of pretreatment with different amounts of folates. Data represent the means ± S.D. of five separate experiments. ^*∗*^ *p* < 0.05.

SOCS1 and SOCS3 expression was detected at basal levels in unstimulated cells. Conversely, LPS treatment determined a significant downregulation of the expression levels of SOCS proteins in BV-2 cells in comparison to control, according to that reported in literature [14]. Cell treatment with folic acid alone increased, in a dose-dependent manner, both SOCS1 and SOCS3 expression levels in BV-2 cells. Interestingly, folic acid pretreatment before LPS stimulation was able to prevent the LPS-induced inhibition, as reported in Figure 6(a). Finally, the qPCR analysis revealed a significant increase of SOCS1 and SOCS3 transcripts in LPS-activated BV-2 cells submitted to folate pretreatment (Figure 6(b)).

## 4. Discussion

In this study we show, for the first time, that folic acid pretreatment was able to polarize proinflammatory responses to anti-inflammatory responses in LPS-activated microglia cells, blocking the activation of NF-*κ*B and JNK and upregulating p38 MAPK phosphorylation. The modulation of signaling pathways may also be related both to a potent inhibitory effect of folic acid on the expression of iNOS, as well as of NO, IL-1*β*, and TNF-*α* production induced by LPS, and to an upregulating action on anti-inflammatory cytokine IL-10 release in activated microglia. Finally, we also demonstrated that folic acid was able to dose-dependently upregulate SOCS proteins expression in microglia cells.

The hallmark of neuroinflammation is the activation of microglia and the production of cytokines and inflammatory mediators including NO, TNF-*α*, IL-1*β*, IL-6, and INF-*γ*, which can trigger neuronal damage [9, 15–17]. In the substantia nigra of Parkinson's diseases patients an increased density of microglial cells expressing iNOS has been observed [18], which is considered responsible for NO derived neuronal toxicity [19, 20]. In addition, proinflammatory cytokines, such as TNF-*α*, IL-1*β*, and INF-*γ*, are described to cause a potent activation of iNOS [21]. In this context, the deletion of inflammatory iNOS using gene targeting has been shown to have a neuroprotective effect in MPTP-treated mice [22, 23]. Interestingly, our findings demonstrated that folic acid treatment significantly inhibited in a dose-dependent manner NO, TNF-*α*, and IL-1*β* production in LPS-activated BV-2 cells. In addition, reduced NO production was dose-dependently modulated by folic acid through a downregulation of iNOS expression.

Several intracellular signal molecules are involved in the regulation of the inflammatory responses, such as the MAPKs, a group of serine/threonine protein kinases comprising three subfamilies: the p42/p44 ERKs, JNKs, and the p38 [24, 25]. MAPK signaling pathways regulate a variety of cellular activities such as proliferation, differentiation, apoptosis, survival, and inflammatory responses [26, 27]. MAPKs can be activated by various extracellular molecules, such as LPS, leading to the activation of transcription factors, including NF-kB which orchestrates the induction of many inflammatory cytokines [28–30]. In this regard, our results showed that folic acid was able to dose-dependently downregulate JNK phosphorylation in LPS-stimulated cells. Similar effects have been reported on RAW264.7 macrophages, in which folic acid treatment inhibited LPS-stimulated JNK phosphorylation, resulting in the inhibition of proinflammatory responses [13].

Intriguingly, our results showed that p38 phosphorylation resulted enhanced by folic acid treatment in a dose-dependent manner. Considering the importance of MAPK signaling in the regulation of inflammatory responses we proceeded to investigate the role of p38 in the IL-10 overregulation after folic acid treatment of LPS-activated microglia cells. For this purpose, we examined the effect of the p38 pharmacological inhibitor, SB203580, and observed how IL-10 production was significantly reduced in folic acid treated cells, suggesting a crucial role for p38 phosphorylation in the IL-10 modulation. In this context, the p38 pathway has been reported to be important for IL-10 induction in TLR-activated antigen-presenting cells [31] as well as in LPS-stimulated B cells [32].

IL-10 is a potent anti-inflammatory cytokine able to inhibit the production of endotoxin-inducible proinflammatory cytokines such as TNF-*α*, IL-1*β*, and IL-6 in mononuclear phagocytes [33]. It has been shown that IL-10 interferes with the activation of NF-kB, and this activity has been correlated with its ability to block the synthesis of other proinflammatory cytokines [34]. Then, it is possible to postulate that, in our in vitro model, folic acid treatment of BV-2 cells determined a p38-dependent increase of IL-10, which in turn was able to block NF-kB activation.

IL-10 has also been shown to induce the expression of SOCS1 and SOCS3 and this upregulation is thought to be one of the mechanisms by which IL-10 mediates its downstream effects [11, 35]. Moreover, it has been reported that IL-10 induces the expression of SOCS3 in monocytes [36]. In addition, forced expression of the SOCS3 gene in myeloid cell lines markedly inhibits cytokine-induced activation of the JAK/STAT signaling pathway, indicating that IL-10 may inhibit the production of proinflammatory cytokines, as observed in monocytes [10].

SOCS1 and SOCS3 have received increasing attention due to their pivotal roles in regulating immune responses, which result from the fatal effects of a deficiency of either protein [37, 38]. It has been reported that both SOCS1 and SOCS3 directly bind NF-*κ*B p65, leading to its proteolysis and the suppression of NF-*κ*B activation [39–41]. Interestingly, SOCS1 and SOCS3 have also been shown to regulate M1 and M2 macrophage polarization [42, 43]. In this regard, it was observed that SOCS1 is necessary to limit the M1 phenotype in response to IFN-*γ*/LPS stimulation. SOCS1-deficient mice resulted hypersensitive to LPS signaling, as shown by an enhanced I*κ*B*α* phosphorylation demonstrating the role of SOCS1 as a negative regulator of TLR-4 signaling [44].

Similarly to M1 macrophage, microglia cells also respond to insults by transiently activating the release of TNF-*α*, NO, and other pro-inflammatory mediators in order to eliminate pathogenic insults [45]. Prolonged or chronic M1-polarized microglial activation causes the disruption of the blood–brain barrier, leukocyte recruitment, a leukocyte and lymphocyte influx [46], and typical hallmarks of neurodegenerative diseases [47, 48]. To ensure wound healing, microglia must rapidly return to a resting M2-like state, attenuating the inflammatory response and assuring homeostasis. M2-polarized microglia secrete anti-inflammatory factors, including IL-10, as well as expressing SOCS proteins. Failure to shift to a M2 microglia phenotype or a predominance of M1-polarized microglia is associated with worsening tissue damage and neuronal loss, suggesting that M2-polarized microglia act as gate-keepers against neuroinflammation [46, 49–51].

In vivo SOCS1 overexpression in oligodendrocytes suppresses deleterious actions observed in experimental autoimmune encephalomyelitis, as well as attenuating SOCS1 expression in microglia-promoted proinflammatory M1-like microglia and worsening neuroinflammation [52], suggesting that SOCS1 plays a crucial role in modulating cytokine responses and might be a promising therapeutic modulator in CNS disease states. Alterations in SOCS3 expression have also been described in animal models of neuronal injury. SOCS3 expression resulted enhanced in rat cerebral cortex following a cortical impact injury and in the hippocampus following lithium-pilocarpine-induced seizure [53, 54]. These observations underline the importance of SOCS1 and SOCS3, among the SOCS family members, in regulating innate immune responses in CNS [55].

**Figure 7:**
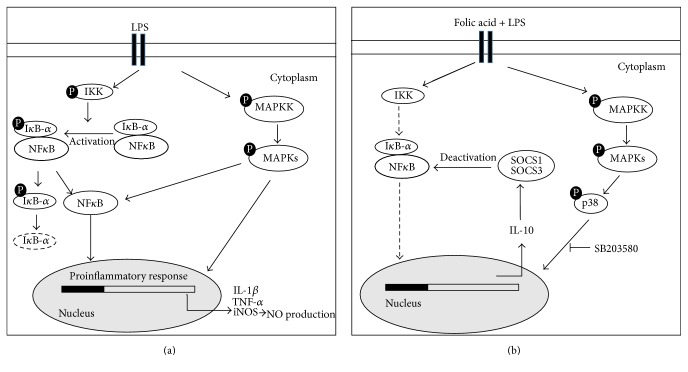
Schematic representation of a probable signaling pathway of folic acid mediated modulation of inflammatory response in microglia cells. (a) LPS activated BV-2 cells increases the expression of proinflammatory cytokines by MAPK phosphorylation and NF-*κ*B activation. The phosphorylation of IKK induces proteasomal degradation of I*κ*B-*α*, enabling the active form of NF-*κ*B to translocate into the nucleus and induce IL-1*β*, TNF-*α*, and iNOS expression. Alternatively, NF-*κ*B is also activated by MAPK phosphorylation triggered by LPS treatment. (b) The folic acid pretreatment of LPS stimulated cells induces p38 MAPK phosphorylation leading to the upregulation of IL-10 production, selectively reduced in presence of p38 pharmacological inhibitor SB203580. IL-10 overproduction increases SOCS expression. Finally, SOCS proteins bind NF-*κ*B and suppress its activation.

Although in literature a number of reports have described the anti-inflammatory effects of folic acid, there is a paucity of information regarding the modulation of SOCS proteins by folic acid in microglia cells [8, 13]. Our study demonstrated that folic acid significantly attenuated the release of proinflammatory mediators in LPS-activated microglia, blocking NF-*κ*B, and overregulating IL-10 dependent SOCS proteins expression through p38 pathways (Figure 7). Thus, we propose that upregulation of SOCS1/3 expression by folate may be an integrated part of the regulatory machinery of inflammatory responses in activated microglia and that at least two distinct pathways may lead to this result. Further in vivo studies of this activity could be useful to investigate the mechanisms involved in the anti-inflammatory actions of folate in order to permit their full exploitation and further explore their promise as therapy in neuroinflammatory diseases.

## Figures and Tables

**Table 1:** DNA sequences of primers used in PCR reactions.

Gene	Sequence (5′ → 3′)	Sequence references
IL-1*β*	FW: GCAGCAGCACATCAACAAGAGC	NM_008361.2
	RW: TGTCCTCATCCTGGAAGGTCCACG	
TNF-*α*	FW: GGCAGGTCTACTTTGGAGTCATTGC	NM_013693.2
	RW: ACATTCGAGGCTCCAGTGAATTCGG	
IL-10	FW: GCCAGTACAGCCGGGAAGACAATA	NM_012854.2
	RW: GCCTTGTAGACACCTTGGTCTT	
ARG-1	FW: TTTCAGGACTAGATATCATGGAAGTG	U_51805.1
	RW: CTTAGGTGGTTTAAGGTAGTCAGTCC	
CD206	FW: GAGGGAAGCGAGAGATTATGGA	BC_141338.1
	RW: GCCTGATGCCAGGTTAAAGCA	
iNOS	FW: CAACAGGGAGAAAGCGCAAA	NM_001313921.1
	RW: TGATGGACCCCAAGCAAGAC	
SOCS1	FW: TGGGCACCTTCTTGGTGCGC	BC_132368.1
	RW: GGCAGTCGAAGGTCTCGCGG	
SOCS3	FW: GCACAGCCTTTCAGTGCAGAG	NM_007707.3
	RW: TTGGCAGCCGTGAAGTCTAC	
GAPDH	FW: ACCACAGTCCATGCCATCAC	BC_085315.1
	RW: TCCACCACCCTGTTGCTGTA	

**Table 2:** Effects of folic acid on cell viability, examined using the MTT test. BV-2 microglial cells were treated with various concentrations of folic acid (FA) (5, 10, 30, 50, and 70 *μ*g/mL) for 24 h. Experimental treatments were analyzed in triplicate and data are expressed as the mean ± SD of five independent experiments.

Folic acid (*μ*g/mL)	% cell viability
0	99.5 ± 0.08
5	98.7 ± 0.15
10	98.2 ± 0.07
30	98.6 ± 0.18
50	98.1 ± 0.05
70	85.3 ± 0.04
